# Traditional Tibetan medicine: therapeutic potential in rheumatoid arthritis

**DOI:** 10.3389/fphar.2022.938915

**Published:** 2022-10-04

**Authors:** Liqiong Yu, Shiling Li, Lili Pu, Chunhong Yang, Qian Shi, Qi Zhao, Shengbu Meniga, Yue Liu, Yi Zhang, Xianrong Lai

**Affiliations:** ^1^ State Key Laboratory of Southwestern Chinese Medicine Resources, School of Pharmacy, Chengdu University of Traditional Chinese Medicine, Chengdu, China; ^2^ State Key Laboratory of Southwestern Chinese Medicine Resources, School of Ethnic Medicine, Chengdu University of Traditional Chinese Medicine, Chengdu, China

**Keywords:** rheumatoid arthritis, traditional Tibetan medicine, bioactive components, immunomodulation, perspectives

## Abstract

Rheumatoid arthritis (RA) is a severe inflammatory autoimmune disease characterized by the failed spontaneous resolution of inflammation. The induction of immune regulation and resolution of inflammatory pathways are effective in alleviating inflammation in RA. As the oldest medical system in the world, traditional Tibetan medicine (TTM) has a long history of preventing and treating RA. This review provides a comprehensive overview of medicinal plants with anti-RA activity in the TTM system, using classic books of Tibetan medicine, modern research literature, and drug standards. A total of 27 species have been found to be effective in treating RA, including *Tinospora sinensis* (Lour.) Merr., *Terminalia chehula* Retz., *P. hookeri* (C. B. Clarke) Hock.), and *Aconitum pendulum* Busch. Alkaloids, flavonoids, polyphenols, and terpenoids have turned out to be the major bioactive components for RA treatment. The inhibition of pro-inflammatory cytokine expression by mediating the NF-κB, MAPK, and JAK/STAT pathways is the core mechanism in RA treatment. In conclusion, this review provides key information and research perspectives for further research on the anti-RA effects of TTM.

## 1 Introduction

Rheumatoid arthritis (RA) is a chronic autoimmune disease that mainly affects the lining of the synovial joints, bones, muscles, blood vessels, and related soft tissues or connective tissues. If the inflammation fails to resolve spontaneously, the disease will remain in patients throughout their lives (Deane and Holers, 2021). The early stages of RA tend to affect small joints, and as the disease progresses, symptoms can appear in the wrists, ankles, and knees, leading to irreversible joint disability, loss of function, and ultimately, considerable disability or premature death (Koga et al., 2021). Approximately 0.1–2.0% of the population worldwide suffered from progressive joint damage, permanent disability, or even shortened lifespan because of RA (Almutairi et al., 2021). In China, the prevalence of RA is higher in Tibet (4.86%) than in other areas of China (Zhang H. et al., 2020).

Many classic well-established agents (e.g., NSAIDs, DMARDs, and glucocorticoids) have their own limiting effects and adverse reactions, such as nausea, vomiting, abdominal discomfort, liver damage, and nephrotoxicity (Hoes et al., 2010; Thakur et al., 2018). Novel classes of biological agents, including TNF-α antagonists and IL-1 antagonists, provide a favorable opportunity for anti-RA with a lower risk of side effects and remarkable treatment results (Ding et al., 2015). However, these drugs are far more expensive than conventional ones, seriously aggravating financial burdens on patients. Thus, seeking inexpensive, effective, and safe anti-RA agents has been an area of great interest.

Owing to their unique geography, people in Tibetan areas are more prone to RA than people in other parts of China. Traditional Tibetan medicine (TTM) has a history of more than 3,800 years of understanding rheumatic diseases and corresponding treatments. To date, 3,105 kinds of natural medicines have been recorded in the Tibetan medicine system (Pan et al., 2021). Tibetan medicine has accumulated a wealth of experience in the treatment of chronic diseases, such as rheumatism, high-altitude polycythemia, cholecystitis, hepatitis, and gastritis (Fu et al., 2020). In fact, Tibetan medicine has been widely used in RA treatment, but these records are fragmented and lack a comprehensive and standardized summary. This situation is not conducive to the development of TTM for RA treatment. This study summarizes the traditional experience and potential value of Tibetan medicine in RA treatment by using a collection of Tibetan medicines for RA treatment from Tibetan medicine classics, such as the “*Dictionary of Chinese Ethnic Medicine*,” “*Drug Standards of Tibetan Medicine*,” “*Chinese Tibetan Medicine*,” and “*Jing Zhu Materia Medica*,” and sorting modern studies on the active ingredients, mechanisms, and therapeutic effects of TTMs for RA.

## 2 Understanding Tibetan medicines for RA treatment

**FIGURE 1:**
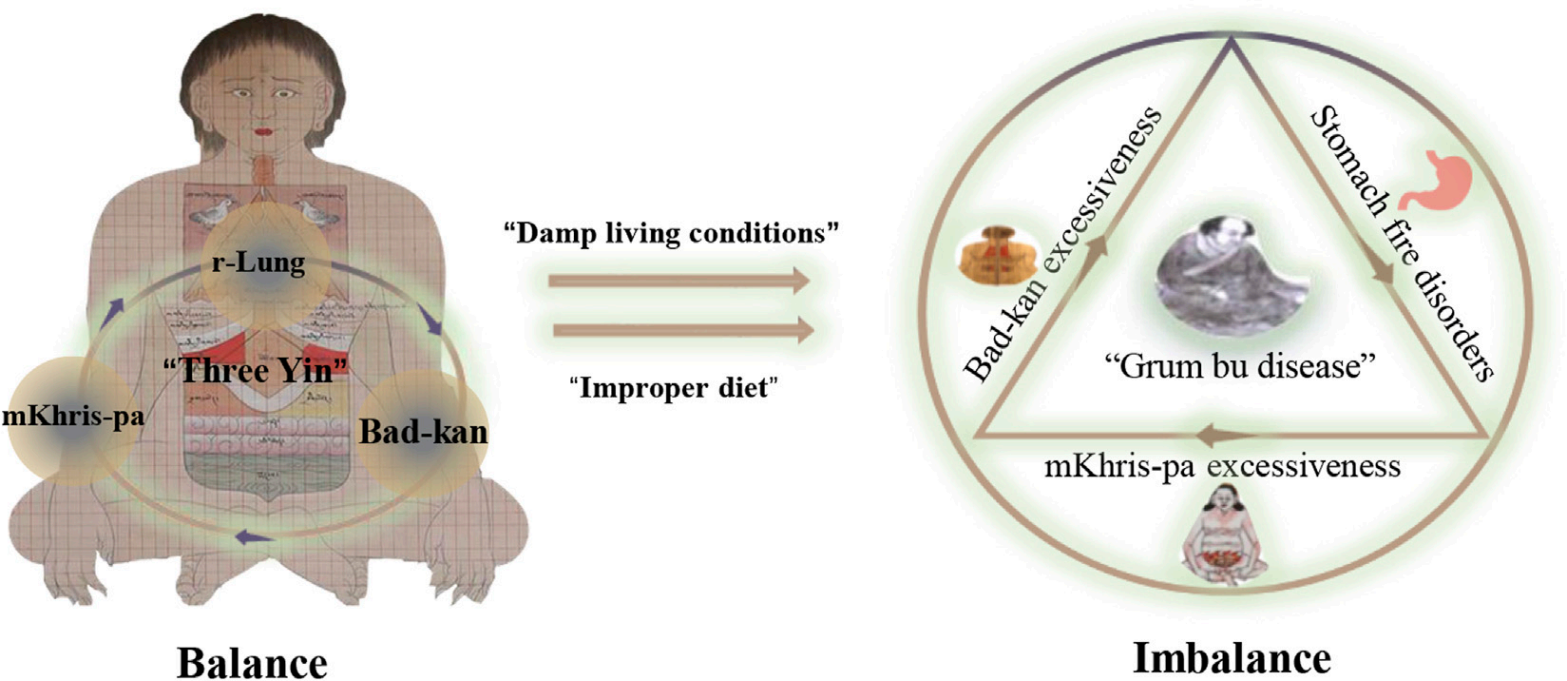
Pathological process of the “Grum bu disease” in Tibetan medicine.

In the theoretical system of Tibetan medicine, RA is regarded as a kind of “Grum bu disease,” which belongs to the category of joint disease and is similar to the “arthralgia disease” of traditional Chinese medicine (Zhijia and Ciren, 2021). According to the Tibetan medical classic “*blue glaze,*” the occurrence and development of the disease is related to three factors (r-lung, mKhris-pa, and Bad-kan). Tibetan medicine states that the disease is due to living in a humid place for a long time and the excessive consumption of fatty, hot, sour, and spicy food, which disrupts the balance of the internal and external environments of the human body and causes stomach fire and dysfunction and the gradual transformation of Bad-kan into mKhirs-pa. Moreover, “yellow water” in the body causes lesions in the meridians, muscles, and joints, resulting in restricted joint movement, stiffness, redness and swelling, and other pathological manifestations of difficult diseases (Ge and Nima, 2021) (Figure 1). “Yellow water” is scattered in the skin, bones, and internal and external organs, exerting nourishing, lubricating, and other physiological effects, nourishing internal organs, and ensuring that the joints move freely. Under the influence of a variety of internal and external pathogenic factors, the human body has three factors of imbalance, resulting in the abnormal quality and quantity of “yellow water,” inducing “yellow water” disease (Bian et al., 2019). In modern medicine, the “yellow water” disease is mainly manifested in RA and skin diseases (Grum bu disease). Owing to the geographical environment, people’s living habits, and other factors in plateau areas, “Grum bu disease” is a common disease. Thus, its treatment has been systematically researched in Tibetan medicine. It is recorded that the treatment of RA mainly focuses on combinations of internal and external treatment and starts from the regulation of diet and living. The combination of Tibetan medicine with medicinal baths, fire moxibustion, and other characteristic external treatment methods, has achieved a unique clinical effect for the treatment of the “Grum bu disease” (Zhijia et al., 2021).

## 3 Methods

This study searched the “*Treasure House of Tibetan Medicine Prescriptions,*” “*Jing Zhu Materia Medica,*” “*Dictionary of Chinese Ethnic Medicine,*” “*Drug Standards of Tibetan Medicine,*” “*Tibetan Medicine Annals,*” and other Tibetan medicine monographs and drug standards, to obtain information on TTMs and their effects against RA. The names, original species, families, medicinal parts, and treated diseases were listed in detail. Plant names were mainly derived from references and verified by their Chinese names in the “Flora of China” (http://frps.eflora.cn/) and the “Medicinal Plant Names Services: Royal Botanic Gardens” (https://www.kew.org/). At the same time, this study performed a large-scale text mining of online Chinese databases (e.g., CNKI, Wanfang, and Weipu) and international databases (e.g., NCBI, Open Access Library, Science Direct, and Google Scholar) to obtain information about the active ingredients of Tibetan medicines for RA and their biological or pharmacological effects.

## 4 TTMs with anti-RA effects

Studies on Tibetan medicines with anti-RA activities were reviewed. A total of 27 species of Tibetan medicinal plants were associated with RA treatment. The Latin names, Tibetan names, medicinal parts, and active parts of relevant modern research of these 27 Tibetan medicines for anti-RA are listed in Table 1. This Tibetan medicine is distributed in 18 families. The most common families are Gentianaceae, Ranunculaceae, and Asteraceae. Furthermore, among the plant parts, the most frequently used medicinal parts are whole grass, roots, leaves, and flowers. Alkaloids, flavonoids, phenolic acids, and other chemical components are the main materials responsible for the anti-RA effects of these TTMs. Immune-regulatory cytokines are important factors for anti-inflammatory effects in RA. The following sections will focus on the mechanisms related to immunomodulatory cytokines and anti-inflammation associated with the treatment of RA with TTMs and their compounds.

**TABLE 1:** Tibetan medicine for anti-RA (Tibetan medicine names are ranked from high to low in the frequency of use).

Medicine	Tibetan name	Family	Medicinal parts	Active ingredients	Traditional use	Experimental model	Effective dose	Function	References
*Tinospora sinensis* (Lour). Merr	སླེ་ཧེུས།	Tetrandraceae	Stem	Quercetin	Treating rheumatic pain	CIA model AA model	6 g/kg 4 g/kg	IL-1α, TNF- α, IL-6↓	(Zeng et al., 2017; Xiong et al., 2019)
*Terminalia chehula* Retz	ཨ་རུ་ར།	Combretaceae	Fruit	Chebulanin	Eliminating dampness	CIA model	80 mg/kg	TNF-α, IL-6, COX-2, MMP-3↓	(Liu et al., 2017; Liu et al., 2019)
*Codonopsis thalictrifolia* Wall.var.mollis Chipp	ཀླུ་བདུད་རྡོ་རྗེ།	Campanulaceae	Whole grass	Flavonoids	Treating rheumatoid arthritis	CIA model	1.76 g/kg	NF-κB, IL-1β, TNF- α, IL-6↓	Zhao et al. (2021a)
*P. hookeri* (C. B. Clarke) Hock.)	སྤ་རྩེ་དོ་བོ།	Dipsacaceae	Whole grass	Bis-iridoids	Relieving arthritis pain	HEK293 cell	100 μL	NF-κB, TNF-α↓	Chen et al. (2018)
*Aconitum pendulum* Busch	ཐོང་ང་ནག་པོ།	Ranunculaceae	Root tuber	Total alkaloid	Treating rheumatoid arthritis	AA model	40 mg/kg	IL-lβ, TNF- α↓	Zhang et al. (2013)
*A.flavum* Hand. -Mazz	ཐོང་ང་ནག་པོ།	Ranunculaceae	Root tuber	3-Acetylaconitine	Curing rheumatism	CIA model	0.3–0.5 mg/kg	Inhibiting the swelling, carrageenan-induced edema	Jain et al. (2021)
*Achyranthes bidentata* Bl	སྲེ་ལོང་།	Amaranthaceae	Root	Total saponins	Relieving rheumatoid arthralgia and myalgia	CIA model AA model	120 ug/g	IL-2, IL-6, TNF-α↓	Song et al. (2020)
*Astilbe rivularis* Buch. -Ham. Ex D. Don var. myriantha (Diels) J.T.Pan	རྒྱ་རྩི་དུག་ལོ་དམར་པོ།	Saxifragaceae	Rhizome	Rhizome glycosides	Treating rheumatism pain	CIA model	5 mg/kg	Symptom scores↓	Ma and gao., (2018)
*Gentiana macrophylla* Pall	ཀྱི་ལྕེ་ནག་པོ།	Gentianaceae	Flowers or whole grass	Ethanol extract	Dispelling wind dampness and stopping arthralgia	AA model	2.5 g/kg	IL-6, TNF-α, p-JAK2, p-STAT3↓, IL-10↑	Yang et al. (2018)
*G. crassicaulis* Duthie ex Burkill	ཀྱི་ལྕེ་ནག་པོ།	Gentianaceae	Flower	Polysaccharides	Curing rheumatoid arthritis	RAW264.7 cell	200 μg	IL-1β, NO, TNF-α↓	Yu et al. (2021)
*G. straminea* Maxim	ཀྱི་ལྕེ་དཀར་པོ།	Gentianaceae	Flowers or whole grass	Alcohol extract	Ameliorating rheumatoid arthritis	CIA model	25 mg/kg	IL-1β, IL-6, TNF-α, NF-κB↓	Jia et al. (2018)
*Erigeron breviscapus* (Vant.) Hand-Mazz	-	Asteraceae	Whole grass	Total flavonoids	Treating rheumatic pain and paralysis	AA model	10 mg/kg/d	TNF-α↓	(Wu and Yang, 2003; Ji and Chen, 2005; Li et al., 2013)
*G. dahurica* Fischer	སྐྱི་རྒྱེ་ནག་པོ།	Gentianaceae	Flowers or whole grass	Total iridoid glycosides	Curing rheumatoid arthritis	CIA model	30 mg/kg	TNF-α, IL-1β, IL-6↓, and regulating the expression of iNOS and COX-2	Jia et al. (2016)
*G. officinalis* H.Smith	ཀྱི་ལྕེ་	Gentianaceae	Flowers	Total iridoid glycosides	Dispelling wind and eliminating dampness	AA model	100 mg/kg	TNF-α, IL-1β, IL-6, MMP1, MMP3↓	Shen et al. (2018)
*Geranium wilfordii* Maxim	-	Ranunculaceae	Whole grass	Whole grass	Eliminating dampness	AA model	-	VEGF↓, TGF-β1↑	Wan et al. (2014)
*Pseudognaphalium affine* (D. Don) Anderberg	གམྤྟ་ བྷ་ཏུ།	Asteraceae	Whole aboveground grass	Alcohol extract	Curing rheumatism	CIA model	0.4 g/kg	NALP3, XOD UA, IL-1β, TNF-α↓	(Wang et al., 2019; Huang et al., 2020)
*Lapotra bulbifera* (Sieb.et Zucc). Wedd	ཟྭ་ལོ།	Urticaceae	Leaves	Ethyl acetate extract	Alleviating arthritis pain	CIA model	17.5 mg/100 g	GATA-3↑	(Sun et al., 2008, Han. 2018)
*Psammosilene tunicoides* W. C. Wu et. C. Y. Wu	-	Caryophyllaceae	Root	Total saponins	Treating rheumatism pain	CIA model	0.15 μg/mL	NLRP3, IL-1β, IL-18, IL-6, TNF-α.↓	He. (2021)
*Saposhnikovia divaricata* (Turcz.) Schischk	ཏང་ཀུན་དཀར་པོ་	Apiaceae	Root	Petroleum ether extract	Treating wind–cold–dampness arthralgia	CIA model	220 mg/kg	IL-1β, IL-6, TNF- α, PGE2↓	Song et al. (2021)
*Angelica dahurica* (Fisch. ex Hoffm). Benthet Hook. f. ex Franch. et Sav	དབྱི་མོང་སེར་པོ་	Apiaceae	Root	Volatile oil	Dispelling wind and eliminating dampness	AA model	140 mg/kg	NOS, NO, TNF-α, PGE2↓	Sun et al. (2016)
*Xanthium strumarium* L	བྲིཚེར།བྲི་ཚེར།་	Asteraceae	Aboveground part	Phenolic	Curing rheumatism pain	AA model	300 mg/kg	COX-2, 5-LOX, TNF-α, IL-1β↓ IL-10↑	(Lin et al., 2014) Jia et al. (2016)
*Lycopodium* Clavatum L	ཆུ་སྲིན་སྡེར་མོ།	Lycopodiaceae	Whole grass	Alkaloid	Dispelling wind and eliminating dampness	AA model	8 mg/kg	ICAM-1, MMP-3, OPG, RANKL/OPG, RANKL↓	(Zhang et al., 2020b; Wang et al., 2021; Zhang et al., 2021)
*Xanthoceras sorbifolia* Bunge	ཙནྡ་སེང་ལྡེང་།	Sapindaceae	Stem and branch	Ethanol extract	Ameliorating rheumatoid arthritis	CIA model	1.35 g/kg	IL-1, TNF- α↓	(Kung and Liu, 2002; Chen et al., 2021a; Jia et al., 2021)
*Sophora flavescens* Ait	སླེ་ཏྲེས།	Fabaceae	Stem	Alkaloid	Treating rheumatoid arthritis	CIA model	50 mg/kg	TNF-α, IL-17A, RORγt↓ FOXP3↑	Ma et al. (2017)
*Rhamnella gilgitica* Mansf. et Melch	སེང་ལྡེང་།	Rhamnaceae	Central core of tree	Flavonoids	Treating rheumatism pain	CIA	38.85 mg/kg	IL-1β, IL-6, IL-17, TNF-α, and INF-γ↓IL-4, IL-10↑	Su et al. (2021)
*Lamiophlomis rotate* (Benth.) Kudo	རྟ་ལྤགས།	Lamiaceae	Aboveground part	Flavonoids	Dispelling wind and eliminating dampness	AA	200 mg/kg	TNF-α, IL-1β, IL-6↓	Chen et al. (2021b)
*Oxytropis falcate* Bunge	སྔོ་སྟག་ཤ།	Fabaceae	Whole grass	Flavonoids	Curing rheumatoid arthritis	AA	100 mg/kg	IL-1β, TNF-α, COX-2↓	Gu et al. (2020)

In addition, there are a total of 44 kinds of Tibetan medicine prescriptions for anti-rheumatism recorded in the “Treasure House of Tibetan Medicine Prescriptions” and “Tibetan Medicine Standards.” By reviewing modern pieces of literature, perhaps because of the complex formulations and diverse components of Tibetan medicinal prescriptions, we found only four prescriptions with modern pharmacological research on RA. These are Ershiwuwei Lvxue Pill, Shiwei Ruxiang Powder, Shibawei Dangshen Pills, and Twenty-Five Wei’er Tea Pills (Ding and Zhang, 2017; Zhu et al., 2017; Liu et al., 2021; Li C. Y. et al., 2022). Detailed information of these prescriptions is given in Supplementary Tables S1, S2.

### 4.1 Main components

#### 4.1.1 Alkaloids

**FIGURE 2:**
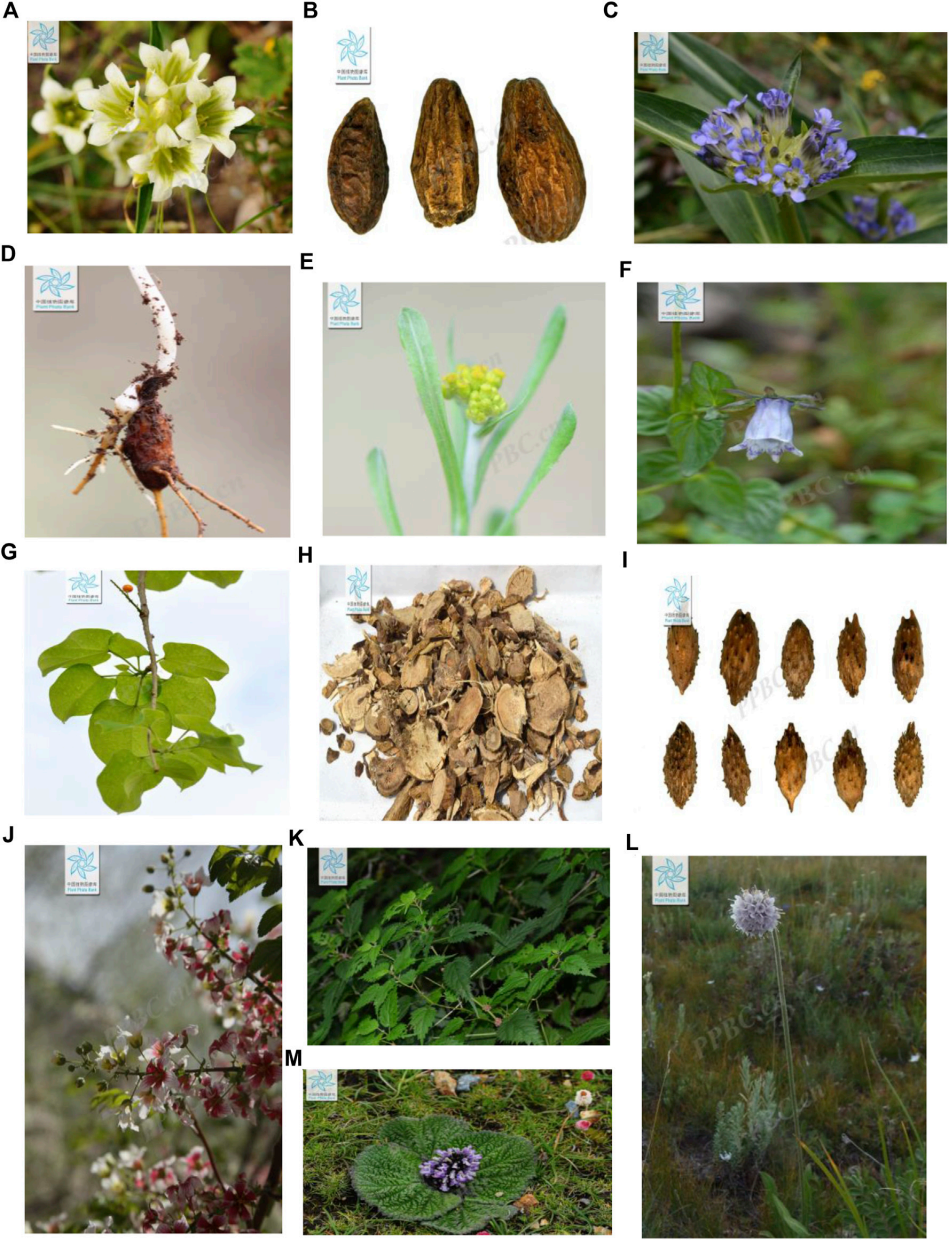
Tibetan medicines are commonly used to treat rheumatism (all the images are collected from the Flora of China, http://ppbc.iplant.cn) **(A)**. **(G)** Maxim. **(B)**. *T. chehula* Retz **(C)**. *G. macrophylla* Pall. **(D)**. *A. pendulum* Busch **(E)**. *P. affine* (D. Don) Anderberg **(F)**. *C. thalictrifolia* Wall. var.mollis Chipp. **(G)**. *T. sinen*sis (Lour.) Merr. **(H)**. *S. flavescens* Ait. **(I)**. *X. strumarium* L **(J)**. *X. sorbifolia* Bunge **(K)**. L. bulbifera (Sieb.et Zucc.) Wedd. **(L)** P. hookeri (C.B.Clarke) Hoeck **(M)**. *L. rotate* (Benth.)Kudo.

**FIGURE 3:**
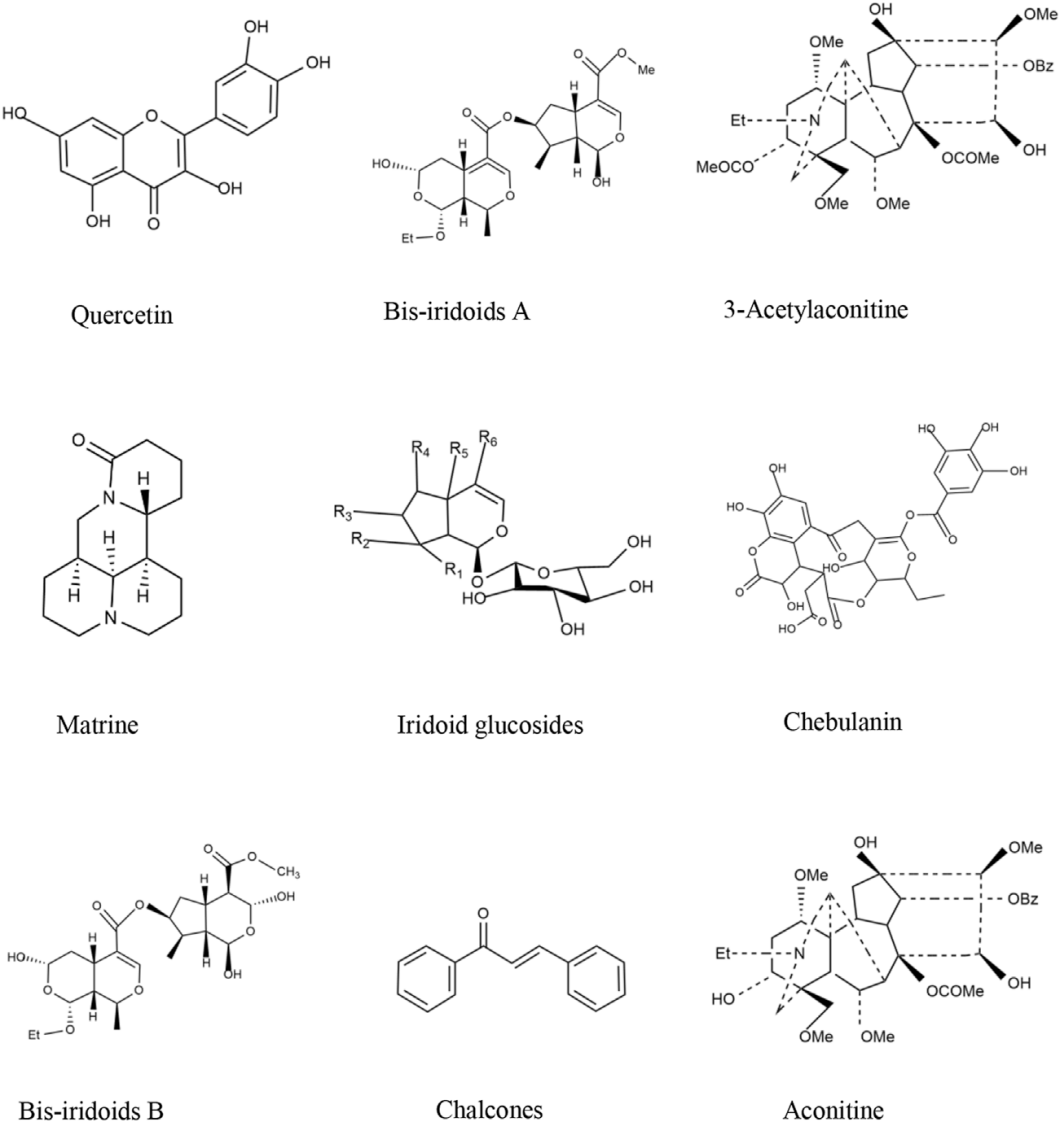
Chemical structures of the main TTM active components with anti-RA effects.

Aconitine (AC) (Figure 3) is the main chemical component in *Aconitum* species, such as the famous TTM *A. pendulum* (Figure 2), which is considered to be the main active substance of Aconitum species and widely applied in the treatment of diverse diseases (Li L. et al., 2022). Low-dose AC (0.3 and 0.9 mg/kg) has good therapeutic potential in heart failure, myocardial infarction, and rheumatic diseases, and can inhibit pain response (Deng et al., 2021).

3-Acetylaconitine (Figure 3) is a nitrogen-containing alkaloid, obtained from *A. pendulum* Busch and A. flavum Hand. -Mazz. (Ranunculaceae) (Li Z. et al., 2022). Pre-clinical research illustrated that 0.03 mg/kg 3-acetyl aconitine combined with Mor or KET has a significant synergic analgesic effect and its mechanism may be associated with NMDA receptors or endogenous opioid peptides (Yao et al., 2014).

Matrine (Figure 3), a tetracyclo-quinolizidine alkaloid, is the main bioactive compound in *S. flavescens* Ait. (Figure 2). Studies have proved that matrine reduces the levels of Th1 cytokines, such as IFN-γ, TNF-α, and IL-1β, and increases the levels of Th2 cytokines (IL-4 and IL-10) to balance the Th1/Th2 axis by regulating the NF-κB signaling pathway (Zhang et al., 2020). Moreover, matrine induces G0/G1 cell cycle arrest and inhibits the activation of the JAK/STAT signaling pathway, thereby increasing the rate of apoptosis *in vitro* (Yang et al., 2017).

#### 4.1.2 Flavonoids

Quercetin (Figure 3) is a natural coumarin compound found in many plants and in some TTMs. It is also the pharmacological basis of *T. sinensis* (Lour.) Merr. (Figure 2). In an AA model, quercetin promoted apoptosis in activated neutrophils. In addition, quercetin inhibited NET formation by suppressing ROS production and autophagy (Yuan et al., 2020). In a recent study, quercetin was shown to inhibit proliferation-induced IL-1 and the expression of matrix metalloproteinases and COX-2 by RA synovial fibroblasts.

Chalcones (Figure 3), 2,4-dihydroxy chalcone, is isolated from TTM *O. falcate* Bunge (Figure 2). (Zhang Q. et al., 2020). It was verified to inhibit NO production in LPS-induced RAW 264.7 cells with 91.91% (10 mol/L). In addition, the anti-inflammatory mechanism of 2,4-dihydroxychalcone may also reduce the production of arachidonic acid by inhibiting cyclooxygenase (COX-1 or COX-2), thus further preventing the synthesis and release of inflammation (Ur Rashid et al., 2019).

#### 4.1.3 Phenolic compounds

Chebulanin (Figure 3) is a natural polyphenol acid isolated from TTM *T. chebula* Retz (Figure 2), which was confirmed to possess anti-inflammatory and anti-arthritic effects by inhibiting the activation of the NF-κB and MAPK signaling pathways in a CIA model (Zhao et al., 2015). In addition, at 100 μM concentration, it could effectively ameliorate RA by inhibiting the nuclear translocation of p38 and p65 in LPS-stimulated macrophages (Liu et al., 2020).

#### 4.1.4 Terpenoids

Iridoid glucosides (Figure 3) widely exist in *L. rotata* (Benth.) (Figure 2) and other TTMs and have strong anti-inflammatory and anti-oxidant activities. Iridoid glucosides (40 mg/kg) could treat RA by inhibiting the production of the serum pro-inflammatory cytokines IL-1β, TNF-α, IL-6, IFN-γ, and IL-17, and increasing the production of the anti-inflammatory cytokine IL-10 (Zhao X. et al., 2021). Iridoid glucosides (30 mg/kg) from *G. macrophylla* Pall. could inhibit the production of TNF-α, IL-1β, and IL-6 and regulate the expression of iNOS and COX-2 (Jia et al., 2016). In addition, bis-iridoids A and bis-iridoids B (Figure 3) from *P. hookeri* (C.B.Clarke) Hoeck. (Figure 2) could reduce the production of NF-κB and pro-inflammatory cytokines such as TNF-α, IL-1β, and IL-6 and down-regulate the expression of inducible nitric oxide synthase and COX-2 in response to lipopolysaccharide stimulation (Chen et al., 2018).

#### 4.1.5 Other kinds of TTM components

Many *in vivo* and *in vitro* studies have confirmed that TTM extracts can exert anti-RA therapeutic effects through different targets. *G. straminea* Maxim (Figure 2) is mainly distributed in Tibet, Sichuan, Qinghai, Gansu, Ningxia, and other places in China at altitudes of 2000–4950 m areas. *In vitro* studies have shown that the ethanol extract of *G. straminea* Maxim (2.5 g/kg) could reduce the levels of IL-1β, IL-6, and TNF-α inflammatory factors, inhibit NF-κB p65 protein expression in synovial tissue, and further reduce synovial inflammation (Wei. 2010; Bao et al., 2018). Similarly, the nontoxic ethanol extract of *G. macrophylla* Pall. (Figure 2) is widely used in the treatment of RA, cholecystitis, and cholelithiasis in China. Europe and other countries showed that it possesses significant anti-inflammatory effects in the AA model, which is demonstrated by inhibiting the expression of IL-6 and TNF-α, up-regulating the expression of IL-10, and down-regulating the relative protein expression levels of p-JAK2 and p-STAT3 (Wang et al., 2013; Lin et al., 2016). *P. affine* (D. Don) Anderberg (Figure 2) is a TTM, whose whole grass on the ground is its medicinal part. Its ethanol extract can alleviate joint inflammation by inhibiting the expression of NALP3 inflammasome-related proteins, restraining the activity of XOD, and reducing the production of UA, IL-1β, and TNF-α in the blood. The ethyl acetate extract of *L. bulbifera* (Sieb.et) Zucc. Wedd. (Figure 3) (5 mg/10 g) showed anti-inflammatory activity by negatively regulating the expression of T-bet in DC and T cells and positively regulating the expression of GATA-3, to affect the balance of Th1/Th2 cytokines in the body and alleviate RA (Wang, He et al., 2019; Huang, Yang et al., 2020). In the CIA model, the ethanol extract of *X. sorbifolia* Bunge (1.35 g/kg) could reduce the levels of IL-17 and TNF-α (Kung, Liu. 2002; Chen, Wu et al., 2021; Jia, Jia et al., 2021). The petroleum ether extract of *S. divaricata* (Turcz.) Schischk. (220 mg/kg) was considered a promising drug for RA treatment because it alleviates inflammatory response and RA symptoms in AA rats (Song, Li et al., 2021).

Apart from that, the combination of glycosides from *A. rivularis* (5 mg/kg) and methotrexate (1.5 mg/kg) could reduce symptoms and morbidity in clinical CIA rats, with no additional side effects and even slightly improved physical changes induced by low doses of MTX compared with monotherapy (Ma, Gao. 2018). The total saponin of *A. bidentata* Bl (120 μg/g) exerts its effect on RA by reversing the imbalance of Th17/Treg and inhibiting the secretion of inflammatory factors (Song, Xu et al., 2020). Similarly, the total saponin (0.15 μg/mL) from *P. tunicoides* could inhibit protein expression of NLRP3 in MH7A cells and reduce the production of inflammatory factors IL-6, IL-18, and IL-1β (Simon et al., 2021).

### 4.2 Pharmacological mechanisms of the anti-RA effect of TTMs

**FIGURE 4:**
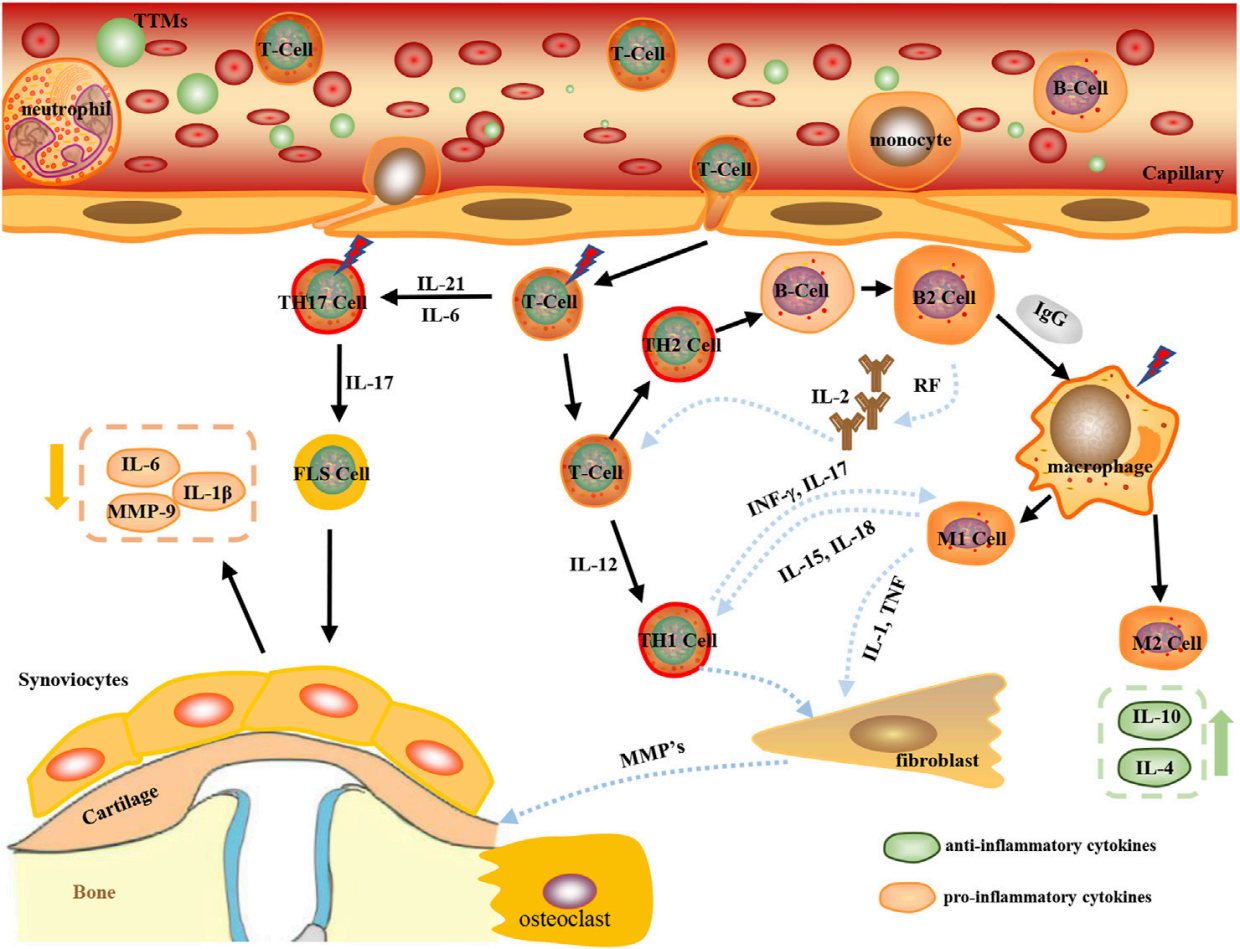
Anti-RA mechanism classification of main TTM active components by immunoregulation.

**FIGURE 5:**
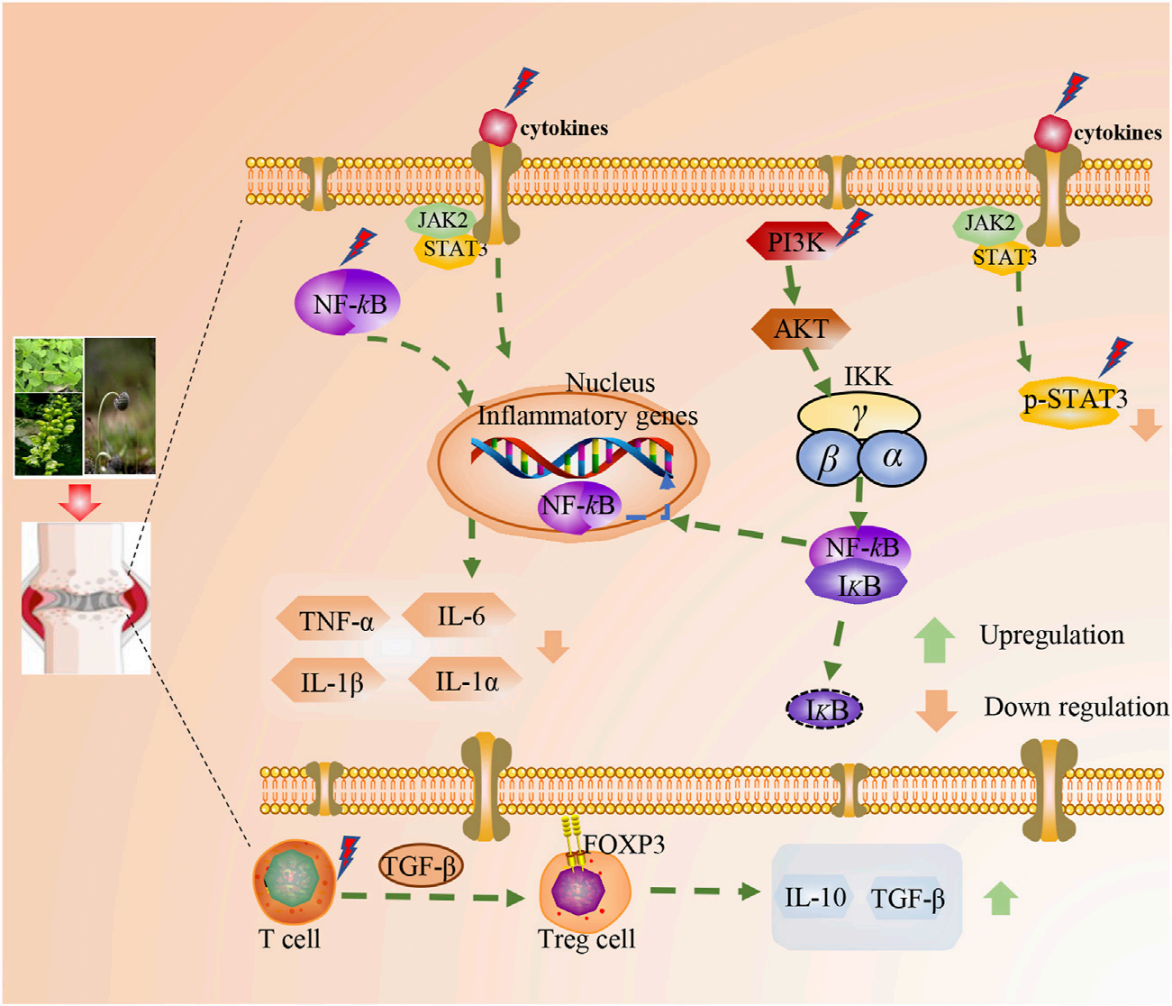
Anti-RA mechanism classification of main TTM active components by anti-inflammation.

The pharmacological mechanisms of TTMs for RA are mainly focused on immunoregulation and anti-inflammation (Figures 4, 5). These pathways do not exist in isolation, but overlap and can influence each other. Cytokines and inflammatory pathways associated with inflammation in RA mainly influence inflammatory response.

#### 4.2.1 Immunopathogenesis of RA

In the inflammatory process of RA, the cascade of innate and adaptive immune responses is an important immunopathogenic mechanism in RA (Chen Z. et al., 2019). This development is driven by an excess of inflammatory cytokines and autoantibodies and maintained by epigenetic changes in fibroblast-like synoviocytes, thus supporting further inflammation (Harre and Schett, 2017). In the process, a large number of different immune cells, including neutrophils, B cells, macrophages, and T cells invade the synovial membrane and fluid. Synovial inflammation reflects subsequent immune activation characterized by the invasion of leukocytes by intrinsic immune cells (e.g. monocytes, macrophages, dendritic cells, and neutrophils) and adaptive immune cells (including Th1, Th2, Th17, and B cells and plasma cell lineage). Th1, Th2, Th17, and regulatory T (Treg) cells differentiate from CD4^+^ T cells (Bystrom et al., 2018; Zhang and Lee, 2018). Treg cells have been extensively studied in several autoimmune diseases (Chen S. J. et al., 2019). Th17 cells represent a distinct effector T-cell subset characterized by the expression of retinoic acid-related orphan receptor (ROR) γt and the production of IL-17 family members, IL-21 and IL-22. Under normal conditions, Th17/Treg cells are in dynamic equilibrium (Astry et al., 2011). It has been identified as a crucial event in the pathogenesis of RA.

In addition, within the innate immunity function that causes inflammation, macrophages play a pivotal role in RA because they are abundant in inflamed synovial tissues and cartilage–vascular opacification junctions (Kinne et al., 2000). They can differentiate into two distinct subpopulations (M1 or M2 phenotypes) with different physiological functions that depend on the microenvironment (Alivernini et al., 2020). M1 cells secrete pro-inflammatory cytokines, while M2 cells secrete anti-inflammatory cytokines. M1 macrophages produce pro-inflammatory cytokines such as TNFα, IL-1β, IL-6, IL-12, IL-23, and low levels of IL-10 and inflammatory enzymes in promoting acute RA (Kennedy et al., 2011). TNF-α is a major pro-inflammatory cytokine critical for immunity to infection. It stimulates inflammation, osteoclastogenesis, and subsequent joint tissue destruction and bone erosion within surrounding joints, which are the main features known to be associated with RA (Radner and Aletaha., 2015). M1 macrophages release inflammatory chemokines, including CXCL13, CXCL9, CXCL5, CXCL10, and CXCL8, to recruit leukocytes to inflammation sites, and these cells produce IL-1β, TNF-αI, IL-6, MMP, chemokine receptors, ROS, and inducible nitric oxide synthase in the joints, leading to joint destruction (Tardito et al., 2019). The main function of M2 macrophages is anti-inflammation. Therefore, in chronic inflammation, M2 macrophages remodel and repair tissues by producing IL-10 and IL-12, expressing CD163 and CD206, and releasing growth factors such as TGF-β and vascular endothelial growth factor (VEGF) (Fassio et al., 2019), (Tanaka et al., 2018). Therefore, reducing the level of pro-inflammatory factors, regulating the dynamic balance of Th17/Treg cells, and promoting the production of anti-inflammatory factors by M2 macrophages are effective methods for RA treatment. Pre-clinical research illustrated that *A. bidentata* Bl, *L. bulbifera* (Sieb.et Zucc.) Wedd, *L. Clavatum* L., and *S. flavescens* Ait. could regulate the imbalance of Treg and Th17 cells by reducing the levels of IL-2, IL-6, and TNF-α in the synovial tissues of CIA or AA model rats. (Yao. 2008, Han., 2018; Ma et al., 2017; Song et al., 2020). Moreover, many TTMs, *T. sinensis* (Lour.) Merr, *T. chehula* Retz, *C. thalictrifolia* Wall. var.mollis Chipp, and *G. dahurica* Fischer regulate cytokines by reducing the level of IL-6 and IL-1. At the same time, *O. falcate* Bunge, *X. sorbifolia* Bunge, *X. strumarium* L., and *P. hookeri* (C.B.Clarke) Hoeck. are potential drugs for RA treatment *via* stimulating the production of anti-inflammatory cytokines, such as IL-10, in M2 cells.

#### 4.2.2 Inflammation-related pathway on anti-RA

A class of proteins originally found in B lymphocytes, NF–κB, can specifically bind to κB sites on various gene enhancers or promoters to initiate gene transcription and plays an important role in cell growth, apoptosis, and inflammatory response (Yin et al., 2015). The NF-κB signaling pathway is a classical pathway regulating inflammatory responses and processes (Wang et al., 2018). At rest, NF-κB p50 binds to the heterodimer formed by p65 and IκB to form an NF-κB-IκB complex, which exists in the cytoplasm in an inactive state (Karwasra et al., 2019). When stimulated by upstream signals, IκB phosphorylation dissociates from the complex and undergoes degradation, inducing the activation of NF-κB p65 through phosphorylation. The activated NF-κB p65 exposes nuclear localization sequences, which can be redirected into the nucleus, bind to specific sites, initiate gene transcription, induce the production and release of a large number of inflammatory factors (such as TNF-α, IL-1β, and IL-6), and trigger an inflammatory cascade response, ultimately inducing the release of inflammatory factors, regulating oxidative stress, and accelerating inflammation progression (Hong et al., 2018; Xia, et al., 2018). NF-κB signaling pathway-related protein levels are significantly elevated in patients with RA and animal models (Li et al., 2020). Therefore, the NF-κB pathway is a major target in the development of therapeutic agents for RA. Modern studies have shown that the ethanolic extract of *C. thalictrifolia* Wall. var.mollis Chipp can significantly reduce the expression levels of p-NF-κB p65 and p-IκB proteins and significantly increase the expression levels of IκB proteins in the synovial tissues of CIA model rats and, thus, alleviates synovial pathological damage (Zhao et al., 2021). The glycosides of *P. h*ookeri (C.B.Clarke) Hoeck can reduce the expression levels of TNF-α, IL-1β, IL-17, and NF-κB in AA rats, effectively reduce the swelling of the inflammatory side of the foot and plantar, and improve pathological changes in the synovial joints of AA rats (Ma et al., 2017; Chen et al., 2018).

In addition, the JAK-STAT signaling pathway is a common pathway for multiple cytokine signaling cascades and is involved in the ligand-induced transcriptional activation of target genes (Xin et al., 2020). The binding of ligands to receptors induces JAK phosphorylation, which in turn promotes STAT phosphorylation, thereby regulating the transcription of target genes encoding pro-inflammatory cytokines and chemokines and directly contributing to RA tissue damage (Choe et al., 2013). STAT3 is activated throughout the course of RA, and the p-STAT3 level increases, which aggravates the inflammatory response by inhibiting FLS apoptosis and promoting T-cell survival and other biological effects (Park et al., 2014). Therefore, the targeted inhibition of p-STAT3 activity is a good research direction. The alcoholic extract (2.5 g/kg) of *G. macrophylla* Pall*.* inhibited the expression of IL-6 and TNF-α, up-regulated the expression of IL-10, and down-regulated the relative protein expression of p-JAK2 and p-STAT3 in the sera of AA rats, thus regulating the JAK2/STAT3 pathway and inhibiting related responses (Wang et al., 2013). In addition, the administration of 900 mg-kg-1-d-1 by gavage can inhibit the level of the anti-CCP antibody and TNF-α in the sera of rats with collagen-induced arthritis at an early stage, reduce the degree of joint swelling, alleviate the symptoms of synovitis, and protect the joints, showing effectiveness in early RA treatment (Lin et al., 2016). Meanwhile, the PI3K/Akt signaling pathway bridges the imbalance between FLS proliferation and apoptosis. The activation of PI3K increased the expression of chemokine SDF-1, thus increasing the number of osteoclast precursor cells and enhancing migration (Dinesh and Rasool., 2018). The expression of TNF-α-induced B lymphocyte-induced maturation protein 1 (Blimp 1) can be significantly inhibited by blocking the PI3K/Akt pathway, and thus, disturbing the PI3K/Akt pathway may be a novel approach for RA treatment. Through network pharmacology and experimental validation, T.sinensis (Lour.) Merr. can disrupt PI3K-Akt signaling pathway and mediate the expression of the major apoptotic genes of RA, such as synovial fibroblasts and B cells, regulate apoptosis, and alleviate RA (Meng, 2020).

## 5 Clinical effects of Tibetan medicine formulas on RA treatment

The Ershiwuwei Lvxue Pill is included in the standard of the Tibetan Medicine Ministry (standard number WS3-BC-0149-95). It dispels wind, removes dampness, and dries yellow water and is thus used to treat arthritis, RA, and gout (Tibet Health Bureau et al., 1979b). Pharmacological studies have shown that the Ershiwuwei Lvxue Pill can reduce the levels of serum pro-inflammatory cytokines (TNF-α, IL-6, and IL-17), increase the level of anti-inflammatory cytokine IL-10, down-regulate the mRNA and protein expression levels of Bcl-2, and up-regulate Bax, SOCS1, and SOCS3 (Liu et al., 2021). A total of 180 cases were clinically randomized into three groups, and case-specific information is provided in Supplementary Tables S3. The blank control group received conventional treatment, whereas the test group received the Ershiwuwei Lvxue Pill and conventional treatment. The positive control group received compound Xuanju capsules and conventional treatment. The course of treatment was 12 weeks in all cases. Blood was drawn and sent for examination before and after treatment, and the time of morning stiffness, number of joint swelling, and number of joint pressure pain were determined. The total effective rate of the test group was 91.67%, while that of the positive control group was 88.3%. The combination of the Ershiwuwei Lvxue Pill with non-steroidal anti-inflammatory analgesic and rheumatism improving drugs for RA can significantly improve symptoms with few adverse effects (Lin et al., 2015).

Included in the standard of the Tibetan Medicine Ministry (standard number WS3-BC-0186–95; Tibet Health Bureau et al., 1979c), the Shibawei Dangshen Pill inhibits the expression of IL-17, NF-κB, and TNF-α in the synovial cells of CIA rats and improves joint inflammation. In addition, it increases the level of caspase-3 and decreases the Bcl-2/Bax ratio in the synovial tissues, thus promoting the apoptosis of synovial cells, inhibiting the abnormal proliferation of synovial membrane, and alleviating inflammation in the joints of CIA rats (Zhu et al., 2017). A total of 88 patients with RA (see Supplementary Tables S3 for case information) received one capsule of the Shisanwei Niaopeng Pill every morning on an empty stomach, three capsules of the Shibawei Dangshen Pill at noon, four capsules of the Twenty-Five Wei’er Tea Pill in the afternoon, and three capsules of the Ershiwuwei Lvxue Pill at night, all consumed with warm water. Dosage was reduced according to the severity of the disease. After the treatment, 55 of the 88 rheumatic patients were cured, and the treatments were effective in 29 cases and ineffective in four cases within 1 year. The total efficiency was 95.45% and no side effects or recurrence were found (Long., 2001).

Shiwei Ruxiang Powder is included in the standard of the Tibetan Medicine Ministry (standard number WS3-BC-0212-095). It dispels wind and reduces dampness and is thus used for eczema, RA, gout, and other rheumatic paralysis and “yellow water” disease (Tibet Health Bureau et al., 1979d). Pharmacological studies have shown that Shiwei Ruxiang Powder can regulate the balance of Th17/Treg cells by decreasing the serum level of IL-17A and increasing the level of TGF-β1 in CIA rats, which in turn can significantly reduce the AI score and paw thickness of swollen limbs in CIA rats to treat RA (Ding and Zhang, 2017). A total of 51 patients with RA were selected for clinical treatment with the Tibetan medicine Shiwei Ruxiang Powder. Specific information about the cases is given in Supplementary Tables S3. Clinical effects, symptoms, and changes in inflammatory factors before and after treatment were observed. The results showed that 31 cases were cured and the treatment was effective in 15 cases and ineffective in five cases. The total effective rate was 90.20%. After treatment, the joint pain index, joint swelling, and morning stiffness were significantly improved compared with those before treatment, and the levels of inflammatory factors such as IL-6, TNF-α, and hs-CRP decreased after treatment (Dou, 2017). The Tibetan medicine Shiwei Ruxiang Powder is effective in the treatment of RA, can quickly relieve clinical symptoms, reduce patients’ pain, and reduce the levels of inflammatory factors.

The Twenty-Five Wei’er Tea Pill is included in the standard of the Tibetan Medicine Ministry (standard number WS3-BC-0141–95). It dispels wind, alleviates paralysis, has anti-inflammatory effects, and causes pain relief. It is used for gout, RA, swollen and painful joint deformation, limb stiffness, and “yellow water” disease (Pharmacopoeia Committee of the Ministry of Health of PRC, 1995). The Twenty-Five Wei’er Tea Pill can decrease the serum levels of TNF-α and IL-6, increase the levels of IL-4 and IL-10, and modulate the pathways of histidine, phenylalanine, alanine, aspartate, and glutamate metabolism. A total of 80 patients with RA were equally divided into control and observation groups. The patients in the control group were treated with diclofenac sodium enteric dissolved tablets orally, whereas the patients in the observation group were treated with 25-year-old tea pills orally. The therapeutic effects of the two groups were observed and compared after one course of treatment. The results showed that the total effective rate of the observation group was 92.5%, while that of the control group was 77.5% (Zha, 2017).

## 6 Conclusion and perspectives

This study summarized the theories of TTM regarding the knowledge and treatment of RA. The results show that the direct cause of RA in TTM is the imbalance of the internal and external environment of the body, which leads to the imbalance and dysfunction of the stomach fire, resulting in the gradual transformation of the Bad-kan prevalence into the mKhris-pa and “yellow water” in the body. Lesions appear in the meridians, muscles, and joints throughout the body (Figure 1). In addition, natural Tibetan medicines traditionally used in the Tibetan system for RA treatment were reviewed. The traditional applications and mechanisms of action of these Tibetan medicines for RA are summarized in Table 1. These Tibetan medicines were mainly distributed in 18 families, and the most commonly used family was Gentianaceae. Alkaloids and flavonoids are the main material bases of Tibetan medicines for RA. Their mechanism of action is mainly the immunomodulation and inhibition of inflammatory factors. The pharmacological effects and clinical outcomes of four Tibetan medicinal formulas for RA treatment were then summarized. Overall, the application of Tibetan medicine to RA treatment is a good strategy with satisfactory clinical efficacy and acceptable safety. Compared with expensive drug discovery models for chemical drugs, a drug screening method that originates from practice used in clinics and guided by the long history of Tibetan medicine theory may be advantageous.

However, the limitations of Tibetan medicine research should be considered. Although TTM has a long history of use in the treatment of RA and sufficient practical experience, modern assessments of clinical effectiveness have not been particularly adequate. For example, the overall efficiency of four Tibetan medicine formulas for the treatment of RA is above 90%, but few cases have been included in the study. Studies on the safety of some drugs are inadequate, and thus, their use may cause problems. For example, the Tibetan medicines *A. pendulum* Busch. and *A. flavum* Hand-Mazz. are drugs with great medicinal value for RA treatment, but at the same time, they are drugs with co-existing toxic effects. Their active ingredient, aconitine, causes poisoning when it exceeds 1 mg (Chan, 2009). These plants are dangerous to use as medicines if not processed and detoxified. Various processing and detoxification methods have been recorded in ancient books (Pu, 2008), such as processing with highland barley wine, frying with sand, and cooking. However, the specific attenuation mechanisms have not been studied in depth. Therefore, detailed studies on these attenuation processes and exploring the laws of material transformation are necessary.

In conclusion, this study provides the first compilation of data for the ethnomedicinal knowledge of TTM in RA treatment. Further studies on TTM will definitely extend its clinical applications. Tibetan medicines have complex formulations and diverse components. Therefore, while conducting scientific research and clinical trials, on the basis of controlling the quality and stability of Tibetan medicine, we should use multiple disciplines to clarify the pharmacodynamic 1basis of the substances and their mechanism of action, and combine novel technologies to explore some directions related to the diagnosis of RA. At the same time, clinical medicine combined with external treatment with Tibetan medicine is a potential and valuable treatment means, for example, medicated bath, acupuncture, and adjustment of diet and daily life.

## Data Availability

The original contributions presented in the study are included in the article/Supplementary Material, further inquiries can be directed to the corresponding authors.

## Abbreviations

RA, rheumatoid arthritis; TTM, traditional Tibetan medicine; CIA, collagen-induced arthritis; AA, adjuvant-induced arthritis; IL-1β, interleukin-1β; TNF-α, tumor necrosis factor-α; NF-κB, nuclear factor kappa-B; MAPK, mitogen-activated protein kinase; JAK/STAT, Janus kinase/signal transducer and activator of transcription; Nrf2, nuclear factor erythroid 2-related factor 2; COX-2, cyclooxygenase 2; MMP-3, matrix metalloproteinase 3; AAc, 3-acetylaconitine; FOXP3, forkhead box P3; RORγt, retinoic acid-related orphan receptor; PI3K, phosphatidylinositol-3-kinase; VEGF, vascular endothelial growth factor; NLRP3, nucleotide-binding oligomerization domain, leucine-rich repeat and pyrin domain-containing 3; NQO1, NAD(P)H quinone oxidoreductase 1; LTB4, leukotriene B4; NOS, nitric oxide synthase; IFN-γ, interferon-γ; NSAIDs, non-steroidal anti-inflammatory analgesics drugs; DMARDs, disease-modifying anti-rheumatic drugs; MTX, methotrexate.
