# Supplementary material for: Traditional Tibetan medicine: therapeutic potential in rheumatoid arthritis
Source: Front Pharmacol. 2022 Oct 4;13:938915. doi: 10.3389/fphar.2022.938915 (PMC9576941; doi:10.3389/fphar.2022.938915)
Supplement: Supplementary file 1 [file DataSheet1.docx]

**Table S1** Tibetan medicine prescription for RA in Tibetan medicine ancient books (Tibetan medicines are listed in alphabetical order)

| **Tibetan medicine prescription** | **Main Components** | **Traditional use** | **References** |
| --- | --- | --- | --- |
| Ershiwuwei Lvxue Pill | *Equus asinus* Linnaeus (50 g), *Tinospora sinensis* (Lour.) Merr (100 g), *Fraxinus rhynchophylla* Hance (80 g), *Gentiana manshurica* Kitag. (80 g), *Pterocephalus hookeri* (C.B.Clarke) Hoeck (70 g), *Rhamnella gilgitica* Mansf. Et Melch (30 g), *Dalbergia odorifera* T.C.Chen (80 g), *Santalum album* L. (50 g), *Terminalia bellirica* (Gaertn.) Roxb (80g), *Terminalia chebula* Retz. (150 g), *Calcareous tuff* (100 g), *Phyllanthus emblica* L. (100 g), *Myristica fragrans* Houtt (30 g), *Eugenia caryophyllata* Thunb. (30 g), *Amomum tsao-ko* Crevost & Lemari´e (30 g), *Amomum kravanh* Pierre ex Gagnep. (30 g), *Cassia obtusifolia* L. (50 g), *Boswellia carteri* Birdw. (50 g), *Gossampinus malabarica* (DC.) Merr (30 g), *Abelmoschus manihot* (L.) Medik (50 g), *Saxifraga pasumensis* Marg.et Shwa. (70 g), Adhatoda vasica Nees. (70 g), *Moschus berezovskii* Flerov. (1 g) *Crocus sativus* L. (10 g), *Bos taurus domesticus* Gmelin (1 g) | Dispelling wind and eliminating dampness | Tibet Health Bureau et al. (1979b) |
| Shibawei Dangshen Pills | *Codonopsis thalictrifolia* Wall.var.mollis Chipp. (150g), *Fritillaria cirrhosa* D. Don (300 g), *Cassia obtusifolia* L. (80 g), *Corydalis gortschakovii* Schrenk. (10g), Brag-Zhun (10 g), *Acorus calamus* L. (40 g), *Tinospora sinensis* (Lour.) Merr (70 g), *Terminalia chebula* Retz. (50 g), G*ymnadenia conopsea* (L.) R. Br. (7.5 g), T*erminalia bellirica* (Gaertn.) Roxb (8.5g), *Moschus berezovskii* Flerov. (5 g), *Boswellia carteri* Birdw. (70 g), *Abelmoschus manihot* (L.) Medik. (70 g), Benzoin (50g), *Acacia catechu* (L.f.) Willci. (70 g), Adhatoda vasica Nees. (70 g), *Phyllanthus emblica* L. (70 g), *Dolomiaea souliei* (Franch.) Shih (75 g) | Relieving pain and inflammation | Tibet Health Bureau et al. (1979c) |
| Shiwei Ruxiang Powder | *Boswellia carteri* Birdw. (100 g), *Terminalia chebula* Retz. (150 g), *Cassia obtusifolia* L. (80 g), *Terminalia bellirica* (Gaertn.) Roxb. (100 g), *Abelmoschus manihot* (L.) Medik. (80 g), *Phyllanthus emblica* L. (120 g), *Dolomiaea souliei* (Franch.) Shih (85 g), *Tinospora sinensis* (Lour.) Merr (100 g), Adhatoda vasica Nees (80 g), Brag-Zhun (10 g) | Dispelling wind dampness and stopping arthralgia | Tibet Health Bureau et al. (1979d) |
| Twenty-Five Wei'er Tea Pills | *Acacia catechu* (L.f.) Willci. (100 g), *Terminalia chebula* Retz. (100 g), *Terminalia bellirica* (Gaertn.) Roxb (125 g), *Phyllanthus emblica* L. (100 g), *Pleurospermum hookeri* var. *thomsonii* C.B.Clarke (50 g), *Polygonatum sibiricum* Delar. ex Redoute (40 g), *Asparagus cochinchinensis* (Lour.) Merr. (40 g), *Oxybaphus himalaicus* Edgew. (25 g), *Tribulus terrestris* L. (30 g), *Boswellia carteri* Birdw (50 g), *Cassia obtusifolia* L. (50 g), *Abelmoschus manihot* (L.) Medik. (35 g), *Tinospora sinensis*（Lour.）Merr. (100 g), *Piper longum* L. (30 g), Fe(processed) (15g), Brag-Zhun (50 g), *Aconitum pendulum* Busch (40 g), Berezovskii Flerov. (1 g), *Acorus calamus* L. (50 g), *Dolomiaea souliei* (Franch.) Shih (50 g), cornu bubali (15 g), concha margaritiferallsta (25 g), *Oxytropis kansuensis* Bunge (40 g), *Rosa sweginzowii* Koehne (50 g), *Gentiana macrophylla* Pall. (30 g). | Dispelling wind and eliminating dampness | Pharmacopoeia Committee of the Ministry of Health of PRC (1995) |

**Table S2** Basic pharmacological data of anti-RA effect of Tibetan medicine prescription

| **Tibetan medicine prescription** | **Experimental model** | **Effective dose** | **Functions** | **References** |
| --- | --- | --- | --- | --- |
| Ershiwuwei Lvxue Pill | CIA | 460 mg/kg | Reducing the levels of serum pro-inflammatory cytokines (TNF-α, IL-6 and IL-17), increasing the anti-inflammatory cytokine IL-10 and down-regulating the mRNA and protein expression levels of Bcl-2, whereas up-regulating Bax, SOCS1 and SOCS3. | (Liu et al., 2021) |
| Shiwei Ruxiang Powder | CIA | 2.2 mg/kg/d | Reducing the levels of IL-17 | (Ding et al., 2017) |
| Shibawei Dangshen Pills | CIA | 90 g/kg/d | Up-regulating the expression of Caspase 3 and down-regulating the ratio of IL-17, NF-KB and Bcl-2/Bax | (Zhu et al., 2017) |
| Twenty-Five Wei'er Tea Pills | CIA | 450 mg/kg/d | Decreasing serum levels of TNF-α, IL-6, increasing level of IL-4, IL-10, and modulating the pathways of histidine metabolism, phenylalanine metabolism, alanine, aspartate, glutamate metabolism | (Li et al., 2022) |

| **Tibetan medicine prescription** | **Number of cases** | **Average age (years)** | **Average duration of illness (years)** | **Formulation** | **Dosage** | **Treatment course (week)** | **Observed indicators** | **Improvement rate (%)）** | **References** |
| --- | --- | --- | --- | --- | --- | --- | --- | --- | --- |
| Ershiwuwei Lvxue Pill | 60 | 48.6 ± 9.21 | 5.96±1.46 | Pills | 0.75 g/tablet, 3 times a day | 12 | Duration of morning stiffness and joint swelling index, ESR, RF | 91.67% | (Lin et al., 2015） |
| Shibawei Dangshen Pills | 88 | 42.5±4.2 | 4.8±2.1 | Pills | Take three pills at noon | 12 | Joint pain index, swelling index, Pressure pain index | 95.45% | (Long., 2001) |
| Shiwei Ruxiang Powder | 51 | 52.6±4.2 | 5.1±1.4 | Powder | 1 g each time, once a day | 12 | Joint pain index, swelling index, Pressure pain index, Duration of morning stiffness, Serum levels of IL-6, TNF-α, hs-CRP | 90.20% | (Dou., 2017) |
| Twenty-Five Wei'er Tea Pills | 40 | 39.4±9.7 | 4.7±2.4 | Pills | Three pills each time, three times a day | 12 | Joint pain index, swelling index, Pressure pain index | 92.5% | (Zha., 2017) |

**Table S3** Clinical efficacy of Tibetan medicine prescriptions in the treatment of RA
